# Behaviors of Perfluorocarbon Emulsion during Dissolution of Oxide Layers

**DOI:** 10.3390/molecules26051329

**Published:** 2021-03-02

**Authors:** Naon Chang, Huijun Won, Chonghun Jung, Seonbyeong Kim, Heechul Eun, Yongsoo Kim

**Affiliations:** 1 Department of Nuclear Engineering, Hanyang University, 222 Wangsimri-ro, Seongdong-gu, Seoul 04763, Korea; yongskim@hanyang.ac.kr; 2 Decommssioining technology research division, Korea Atomic Energy Research Institute, Daedeok-daero 989-111, Yuseong-gu, Daejeon 34057, Korea; nchjung@kaeri.re.kr (C.J.); sbkim@kaeri.re.kr (S.K.); ehc2004@kaeri.re.kr (H.E.)

**Keywords:** perfluorocarbon, emulsion, dissolution, oxide layer, decontamination

## Abstract

This study investigates the dissolution behavior of oxide layers containing radionuclides using perfluorocarbon (PFC) emulsion as a reusable medium. Chemicals such as PFC, anionic surfactant, and H_2_SO_4_ are used for preparing the PFC emulsion, and emulsified using an ultrasonication process. The FTIR results show O–H stretching that is formed by the interaction of the carboxyl group of the anionic surfactant with the hydroxyl group of water containing H_2_SO_4_, and find that the H_2_SO_4_ can be homogeneously dispersed in the PFC–anionic surfactant–H_2_SO_4_ emulsion. The dissolution test of the simulated Cr_2_O_3_ specimen is conducted using PFC emulsion containing KMnO_4_. Through the weight losses of specimens and Scanning Electron Microscope-Energy Dispersive X-ray Spectrometer (SEM-EDS) analysis, it is confirmed that the Cr_2_O_3_ layer on the SUS304 specimen is easily dissolved using PFC emulsion. During the dissolution of the Cr_2_O_3_, it is observed that the dispersed H_2_SO_4_–KMnO_4_ became unstable and separated from PFC emulsion. Based on these results, the behavior of the PFC emulsion during the dissolution of the oxide layer is explained.

## 1. Introduction

Nuclear facilities consist of various metal equipment and metal devices. The metal surfaces can be contaminated with oxide layers containing the radionuclides during the operation of nuclear facilities [1,2]. The contaminated metals increase the amounts of radioactive wastes when they are disposed of without treatment. Therefore, the contaminations on the metal surface are necessary to be removed using various decontamination methods to reduce the amount of radioactive waste generation.

The radionuclides on the metal surface can be removed by dissolving them along with the oxide layer using a chemical solution [2,3]. Although the water-based chemical method is commonly used to dissolve the oxide layer, large amounts of radioactive liquid wastes can be generated. The radioactive liquid wastes are necessary to be treated using UV decomposition or ion exchange resins [3,4]. However, during, the treatment of the radioactive liquid wastes, additional costs are needed, and secondary wastes are generated.

To solve this problem, the methods of using perfluorocarbon (PFC) as a medium for removing the contaminants from the metal surface were studied by various researchers. PFC is the liquid used for cleaning the device, manufacturing the semiconductor, and cooling the electronics [5,6]. PFC is composed of carbon and fluorine, and the chemical formula of PFC is C_x_F_y_. The boiling point, latent heat, and specific heat of the PFC are generally lower than water [7]. The density of the PFC is commonly higher than water. For instance, the density of PF-5058, which is one of the PFC, is 1.75 g/mL [8]. In addition, the PFC has a high radiation resistance [9]. The PFC can be usefully applied to remove the radionuclides from the metal surface due to this feature.

Kaiser studied the removal of the loosely-attached radioactive particles from the various surface by applying the ultrasonication in the bath filled with PFC and surfactant [10]. It was confirmed that 78.0–99.7% of contaminants were removed from the surfaces regardless of the type of specimens [10]. Won studied the removal of the contaminants from the metal surface by spraying the PFC and surfactant mixture [11]. As a result, the radioactive particles were removed from the metal surface because the anionic surfactant reduced the adsorption force between the contaminants and metal [11]. These two methods were the physical method for the removal of the contaminants loosely sticking on the metal surface, thus it cannot be applied to the dissolution of the oxide layer with radioactive contaminant fixed on the metal surface. This is because the PFC medium does not mix with the decontamination agent, reacting with the oxide layer. PFC cannot form a homogenous solution after mixing with the polar molecules. To resolve this problem, it is necessary to mix the PFC with the decontamination agent homogeneously.

In order to homogeneously mix the decontamination agent with the PFC, the emulsification can be applied. The emulsification of the PFC and chemicals has been studied by various researchers. Song studied the cancer therapy method using PFC and hydrophobic TaOx nanoparticles emulsified by an amphiphilic polymer [12]. As a result, the tumor oxygenation and radiotherapy were enhanced by the O_2_ saturation reaction of PFC emulsion [12]. Flögel developed the in vivo visualization of inflammatory processes by magnetic resonance imaging using biochemically inert PFC nanoemulsions [13]. From the above studies, it was found that the PFC emulsion was utilized as a useful medium in the medical field. It was also confirmed that emulsification was effective in mixing the chemicals homogeneously. Therefore, it is expected that the emulsification method can be applied to mix the PFC medium and decontamination agent. In addition, the decontamination agent dispersed in the PFC emulsion can be separated from the emulsion when the emulsion becomes unstable over time. In this case, the used decontamination agent will be located on the PFC because the density of the PFC is higher than the decontamination agent. For this reason, it is expected that the PFC medium can be easily reused after scooping the decontamination agent and the radionuclides dissolved in the decontamination agent.

In this study, an innovative chemical dissolution method of the oxide layer, P-BED (perfluorocarbon-based emulsion for oxide layer dissolution) composed of recoverable PFC, anionic surfactant, H_2_SO_4_, was designed. The objective of this study is to effectively remove the contaminants and the oxide layers from the metal surface using the P-BED medium and to obtain the behavior of the P-BED during dissolution. The chemical structures and stability of the P-BED were analyzed by FTIR. The dissolution performance of the P-BED containing KMnO_4_ was evaluated by dissolution test for the simulated Cr_2_O_3_ layers on the SUS304 specimen.

## 2. Results and Discussion

### 2.1. Characteristics of the P-BED

**Figure 1:**
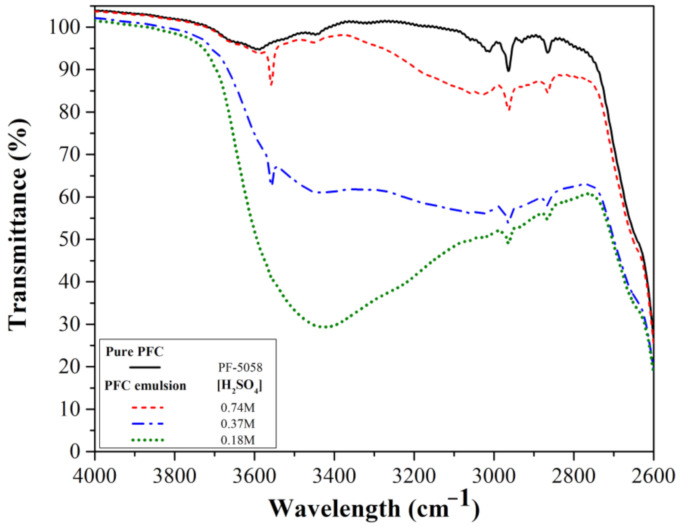
FTIR spectra of the pure perfluorocarbon (PFC) and the P-BED (perfluorocarbon-based emulsion for oxide layer dissolution) consisted of PFC–anionic surfactant–H_2_SO_4_.

Figure 1 shows the FTIR spectra of pure PFC and the P-BED, consisted of PFC–anionic surfactant–H_2_SO_4_ emulsion, under three different concentrations of H_2_SO_4_. As represented in Figure 1, two peaks appeared at 2880 and 2975 cm^−1^ in the pure PFC spectrum and also appeared in the spectra of the emulsions. The two peaks were due to the unique characteristic of PFC. In the three types of emulsions, a sharp peak at 3550 cm^−1^ increased as the concentration of H_2_SO_4_ increased from 0.18 to 0.74 M. Hintze identified the O-H stretching spectra near 3600 cm^−1^ in the vapor-phase sulfuric acid [14]. Therefore, a sharp peak at 3550 cm^−1^, when the concentrations of H_2_SO_4_ were 0.37 and 0.74 M, were considered to be due to H_2_SO_4_. In addition, a broad peak at the range of 3000–3700 cm^−1^ appeared increased when the H_2_SO_4_ concentration decreased from 0.74 to 0.18 M. It was reported that the broad band around 3400 cm^−1^ is associated with the O-H stretching modes of H_2_O [15,16]. Based on this result, we could infer that the interaction of each component in PFC emulsions with the hydroxyl peak of water containing H_2_SO_4_. After emulsification, the carboxyl groups of the hydrophilic head of anionic surfactant (Krytox 157FSM) interact with H_2_O, including H_2_SO_4_, and the hydrophobic tail interacts with PFC [17,18]. Therefore, the broad peak in the range of 3000–3700 cm^−1^ could be attributed to the interaction of different bonds between the hydroxyl group of H_2_O and the anionic surfactant and H_2_SO_4_ when the H_2_SO_4_ concentration was 0.18 M. Because of these results, it can be determined that the P-BED becomes stable because the bond between PFC, anionic surfactant, and H_2_O containing H_2_SO_4_ increases when H_2_SO_4_ concentration decreases from 0.74 to 0.18 M.

**Figure 2:**
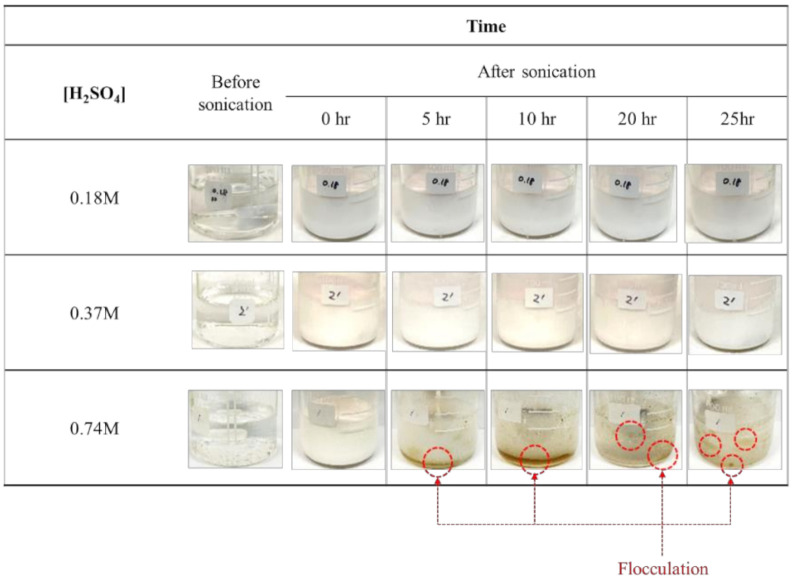
The phase change of the P-BED consisted of PFC–anionic surfactant–H_2_SO_4_ over time.

The phase change of the P-BED with the times was observed to investigate the dispersion stability of the P-BED. The photographs of P-BED are shown in Figure 2. Flocculation was not observed within 25 h when the concentrations of H_2_SO_4_ in the emulsion were 0.18 M and 0.37 M. On the other hand, the flocculation was observed in the P-BED after 5 h when the concentration of H_2_SO_4_ in the P-BED was 0.74 M. It is considered that the flocculation is happened when the emulsion becomes unstable [19]. Therefore, 0.74 M of H_2_SO_4_ was not suitable for the condition of P-BED.

### 2.2. Dissolution Characteristics of the Oxide Layer Using P-BED

**Figure 3:**
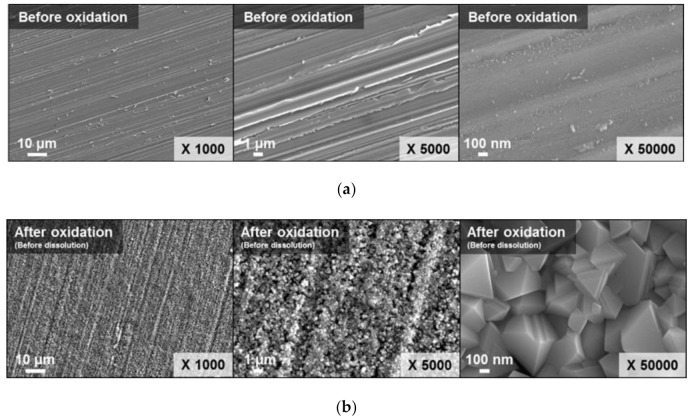
FE-SEM images of SUS304 specimen surface: (**a**) Before oxidation and (**b**) after oxidation.

Before the dissolution test, the simulated oxide layer on the SUS304 specimen was prepared. The surface morphologies of the SUS304 specimen, before and after the high-temperature oxidation, are shown in Figure 3. Figure 3 also shows the crystalline particles formed on the specimen surface after the oxidation. The particles were an octahedral shape, with a diameter of between 100–400 nm, and were aligned along the linear grooves that were formed during grinding. From the Energy Dispersive X-ray Spectroscopy (EDS) result, the oxide layer mainly composed of Cr_2_O_3_, and the content of oxygen of the oxide layer was 61.33%.

Chromium oxide dissolves in an acidic condition, as shown in Equation (1). At 45 °C, the dissolution reaction was calculated by chemical equilibrium calculation program (HSC Chemistry 6.0), and it was confirmed that the reaction proceeds spontaneously. The reaction (1) proceeds as two successive steps, as shown in Equations (2) and (3) [20]. Especially, reaction (3) is known as the Guyard reaction. At the initial dissolution stage, H^+^ ion involves in the dissolution reaction and promotes the dissolution of Cr_2_O_3_. They were also calculated by chemical equilibrium calculation program (HSC Chemistry 6.0).

Cr_2_O_3_ + 2MnO_4_^−^ + H_2_O → 2HCrO_4_^−^ + 2MnO_2_; (△G = −66.56 kcal, at 45 °C)(1)

Cr_2_O_3_ + 1.2MnO_4_^−^ + 0.2H_2_O + 1.6H^+^ → 2HCrO_4_^−^ + 2Mn^2+^; (△G = −39.52 kcal, at 45 °C)(2)

1.2Mn^2+^ + 0.8MnO_4_^−^ + 0.8H_2_O → 2MnO_2_ + 1.6H^+^; (△G = −27.04 kcal, at 45 °C)(3)

**Figure 4:**
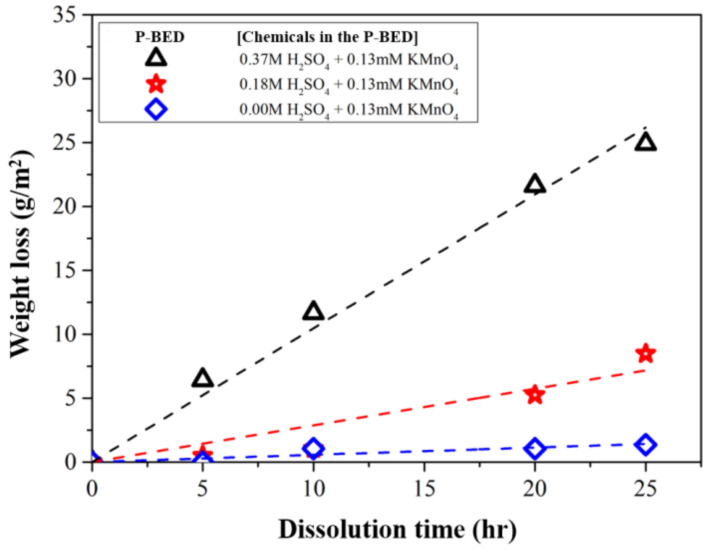
The weight loss of the specimens during the dissolution test for various concentrations of H_2_SO_4_ in the P-BED.

Based on the above dissolution reactions, it was decided to use H_2_SO_4_–KMnO_4_ as the decontamination agent and inject it into P-BED to remove the Cr_2_O_3_ layer during the dissolution test. The effect of the H_2_SO_4_ concentration in the P-BED on the dissolution performance was investigated when the KMnO_4_ concentration was constant at 0.13 mM. As shown in Figure 4, the weight loss of simulated oxide layer specimen increased with increasing H_2_SO_4_ concentration during the dissolution. This result could be explained by the dissolution reaction represented in Equation (2). In particular, the largest amount of Cr_2_O_3_ layer was dissolved when the 0.37 M of H_2_SO_4_ was contained in the P-BED among the experimental conditions.

**Figure 5:**
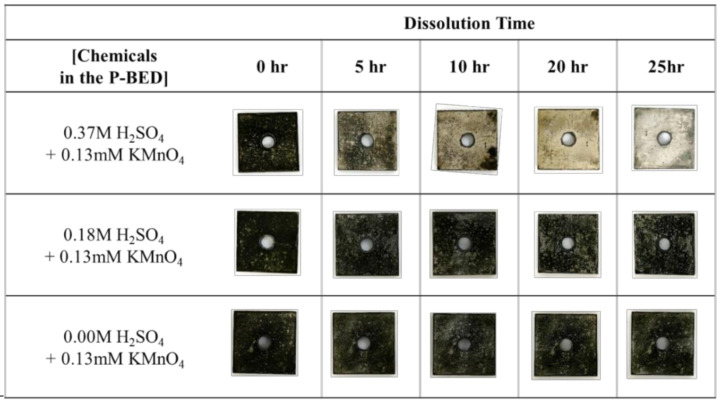
The change in the specimen’s surfaces during the dissolution test for various concentrations of H_2_SO_4_ in the P-BED.

**Figure 6:**
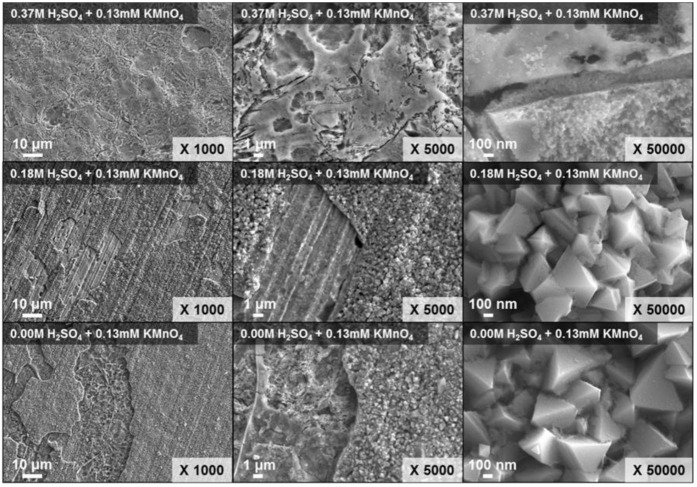
SEM images of the specimens after a 25 h dissolution test under various concentrations of H_2_SO_4_ in the P-BED.

The oxide layers that remained on the specimen were observed after 25 h when the H_2_SO_4_ concentration was lower, as represented in Figure 5. In addition, more oxide layers were removed with increasing the dissolution time. The SEM result was agreed well with the result of the visual test, as illustrated in Figure 6. The more amounts of crystalline particles, which are the oxides, were maintained after the 25 h when the H_2_SO_4_ concentration was lower. In particular, the particles were not observed when the H_2_SO_4_ concentration was 0.37 M. From this result, it was also confirmed that the dissolution performance of Cr_2_O_3_ layers increased when the H_2_SO_4_ concentration in the P-BED was higher. The EDS result of the simulated specimen after dissolution is listed in Table 1. The atomic ratio of oxygen after 25 h of dissolution decreased to 7.96% when the H_2_SO_4_ concentration was 0.37 mM. However, the contents of the oxygen after 25 h of dissolution were 59.44% and 65.96% when the H_2_SO_4_ concentration was 0.18 and 0 mM, respectively. This was because the H^+^ ion was concerned with the dissolution reaction of Cr_2_O_3_. Based on these results, it was determined that the optimized H_2_SO_4_ concentration in the P-BED is 0.37 M for the effective dissolution performance among the experimental conditions. In addition, the above results were compared with the result of the Cr_2_O_3_ dissolution test using ozone conducted by Maroo [21]. They dissolved the Cr_2_O_3_ powder into the solution by injecting the ozone at 50 °C, which is the similar temperature condition of this research. As a result, only 20% of the injected Cr_2_O_3_ were dissolved for 6 h. From this, it was expected that the more amount of Cr_2_O_3_ layer can be removed within the same dissolution time if P-BED is applied compared with application of ozone.

**Figure 7:**
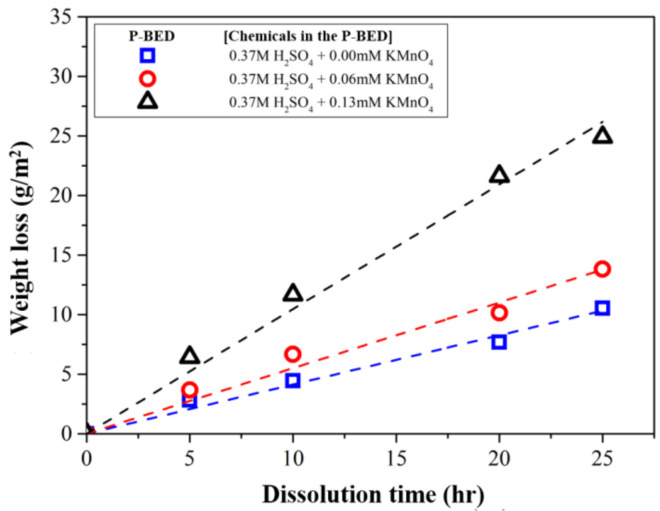
The weight loss of the specimens during the dissolution test for various concentrations of KMnO_4_ in the P-BED.

**Figure 8:**
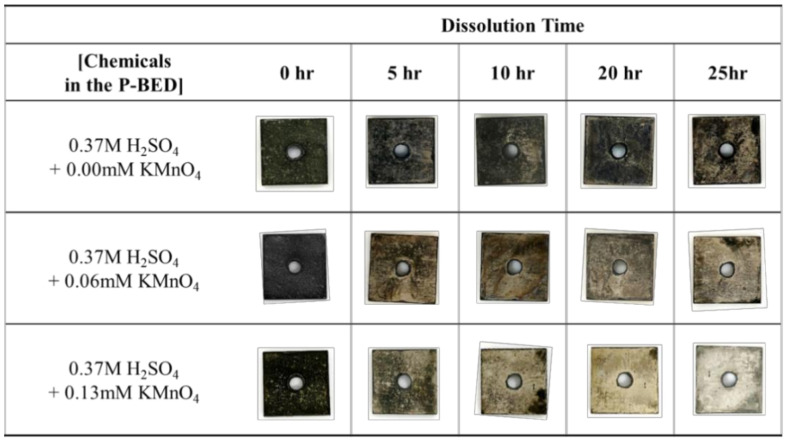
The change in the specimen’s surfaces during the dissolution test for various concentrations of KMnO_4_ in the P-BED.

When the same concentration of H_2_SO_4_ and the various concentrations of KMnO_4_ were added in the P-BED, the weight loss of the simulated specimen over the dissolution time is illustrated in Figure 7. The photographs of the simulated specimens during the dissolution test are shown in Figure 8. As shown in Figure 7, the number of removed oxide layers increased as the concentration of KMnO_4_ increased from 0 mM to 0.13 mM. Because the reaction (2) and (3) increased with increasing the concentration of KMnO_4_. This result can also be found in the photographs of the specimens in Figure 8. Especially, the oxide layers were fully removed after 25 h of dissolution when the KMnO_4_ concentration was 0.13 mM. Based on the result, the optimum concentration of KMnO_4_ in the P-BED for the effective dissolution of the Cr_2_O_3_ layer was determined 0.13 mM among the experimental condition.

**Figure 9:**
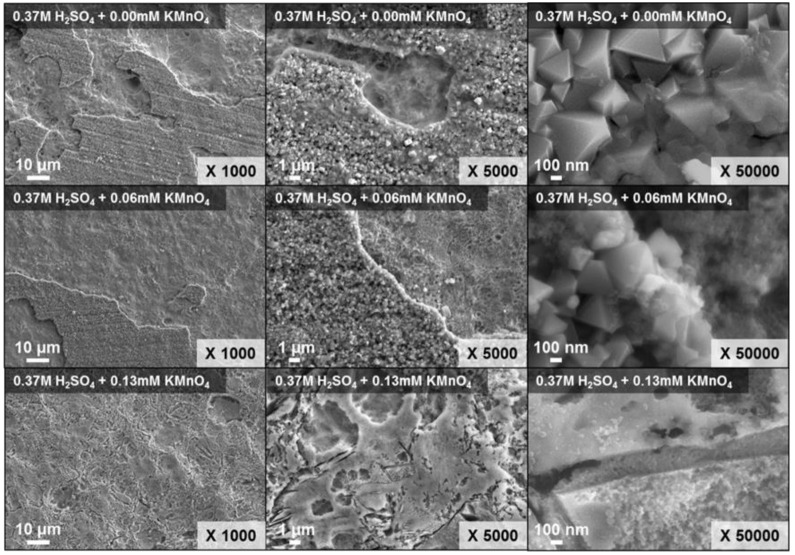
SEM images of the specimens after a 25 h dissolution test under various concentrations of KMnO_4_ in the P-BED.

Figure 9 shows the morphology of the simulated specimens after 25 h of dissolution, when the concentrations of H_2_SO_4_ was 0.37 mM, and KMnO_4_ concentrations in the P-BED was 0 to 0.13 mM. The crystalline particles (which are the oxides) on the specimen decreased with increasing the concentration of KMnO_4_ in the P-BED. EDS results of the simulated specimens after 25 h of dissolution using P-BED was listed in Table 2. The contents of oxygen decreased to 44.17%, 32.97%, and 7.96% after the dissolution when the concentrations of KMnO_4_ in the P-BED were 0, 0.06, and 0.13 mM, respectively. These results were consistent with the results of weight loss, shown in Figure 7, and photographs of specimens, represented in Figure 8. In addition, a part of the oxide layer was removed even when the KMnO_4_ was absent in the P-BED, as shown in Figure 9. Based on the result, it was found that a portion of the oxide layers could also be removed by reaction with H^+^ ions.

### 2.3. P-BED Behaviors during the Dissolution of the Oxide Layer

**Figure 10:**
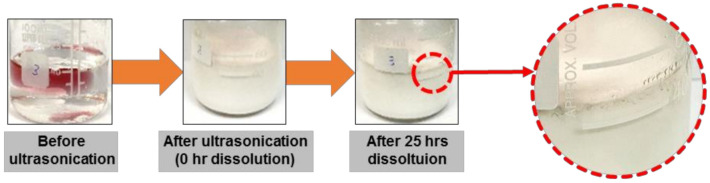
Photographs showing the formation change of the P-BED composed of PFC, anionic surfactant, 0.37 M H_2_SO_4_, and 0.13 mM KMnO_4_ before ultrasonication and during the dissolution test.

Figure 10 shows the photographs of the P-BED consist of PFC, anion surfactant, 0.37 M H_2_SO_4_, and 0.13 mM KMnO_4_ before applying the ultrasonication process and during the oxide layer dissolution test. H_2_SO_4_–KMnO_4_ was injected in the P-BED because they were used as the decontamination agent in the PFC medium. The H_2_SO_4_–KMnO_4_ with a purple color was located at the top of the PFC–anionic surfactant–H_2_SO_4_–KMnO_4_ mixed solution before the ultrasonication. The reason is that the density of H_2_SO_4_–KMnO_4_ was lower than that of PFC, 1.75 g/cm^3^ [6]. According to the pourbaix diagram, the purple color was caused by MnO_4_^−^ ions [22]. After the ultrasonication process, H_2_SO_4_–KMnO_4_ was uniformly dispersed in the PFC, and the P-BED changed to a cloudy phase, as shown in Figure 10. After 25 h of dissolution, the flocculation and the separation of some agents were observed from the P-BED. The flocculated and separated agents were the used decontamination agent. The separated agent did not have a purple color, unlike before the dissolution test. Because the purple MnO_4_^-^ ions in the agent were consumed during the Cr_2_O_3_ removal reactions indicated in Equations (1)–(3). The flocculation and separation of the used decontamination agent, however, were not observed when the dissolution of the oxide layer was not conducted, as shown in Figure 2. Through the results, it was confirmed that the P-BED became unstable as MnO_4_^−^ ions were consumed during oxide layer dissolution.

**Figure 11:**
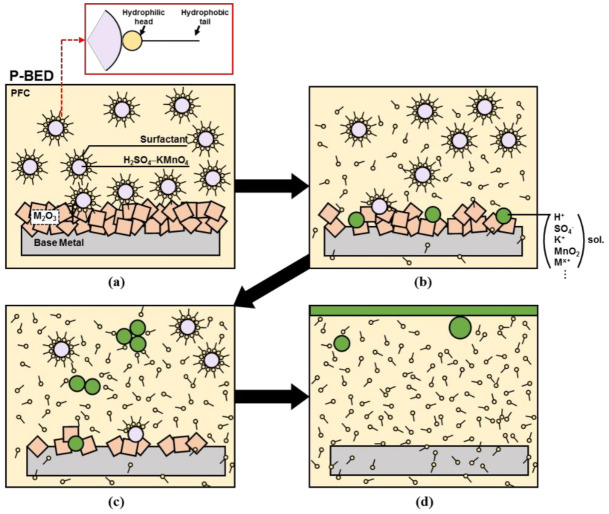
Schematic diagrams of the P-BED behaviors during oxide layer dissolution; (**a**) P-BED after ultrasonication process, (**b**) oxide layer dissolution by reaction with unstable P-BED, (**c**) H_2_SO_4_–KMnO_4_ flocculation after participating the oxide layer dissolution reaction, and (**d**) separation of H_2_SO_4_–KMnO_4_ after finishing the oxide layer dissolution.

Based on the results, the behaviors of the P-BED during the dissolution of the oxide layer were suggested, as indicated in Figure 11. As shown in Figure 11a, the hydrophilic head of the anionic surfactant, which had the carboxyl group, formed an O-H bond with the water in the H_2_SO_4_–KMnO_4_ after the ultrasonication process. In addition, the P-BED was emulsified as the hydrophobic tail of the anionic surfactant interacted with PFC. As seen in Figure 11b, the number of bonds between the anionic surfactant and the water decreased as the P-BED became gradually unstable over time. However, the flocculation did not occur at this time, and the H_2_SO_4_–KMnO_4_ participated in the dissolution reaction of the oxide layer. The bond between the water and the surfactant decreased further over time, and the P-BED offered more activation sites for dissolution reaction. Therefore, oxides were dissolved in the metal ions. The metal ions coexisted with H^+^, SO_4_^−^, and K^+^ ions in the decontamination agent of P-BED. Afterwards, the H_2_SO_4_–KMnO_4_ participated in the dissolution reaction of the oxide layer began to flocculate with each other, as shown in Figure 11c. After finishing the dissolution, the used decontamination agent containing various ions was finally separated, and they were located at the top of the P-BED as represented in Figure 11d. Due to the characteristic of P-BED, the used decontamination agent can be easily removed from the P-BED by scooping method after finishing the oxide layer dissolution. Therefore, the PFC and surfactant mixed solution located at the bottom can be reused for removing the oxide layers after re-injecting the decontamination agent and applying the ultrasonication process.

## 3. Materials and Methods

### 3.1. Materials

P-BED was composed of 93 vol% of PFC (3M, PF-5058, St. Paul, MN, USA), 3 vol% of anionic surfactant (Krytox 157FSM, Dupont, Wilmington, DE, USA) and 4 vol% of a decontamination agent. The total volume of the P-BED is 50 mL, and they were prepared in the bottle with a lid. The decontamination agent consisted of H_2_SO_4_ (98%, DAEJUNG, Siheung-si, Gyeonggi-do, Korea). When the dissolution test was performed, KMnO_4_ (>99.3%, DAEJUNG, Siheung-si, Gyeonggi-do, Korea) was additionally injected into the decontamination agent. DI water (Direct-Q^®^ 3 UV, Millipore, Burlinton, MA, USA) was used for preparing the decontamination agent. The concentrations of H_2_SO_4_ and KMnO_4_ in the P-BED were 0 to 0.74 M and 0 or 0.13 mM, respectively.

### 3.2. P-BED Preparation

**Figure 12:**
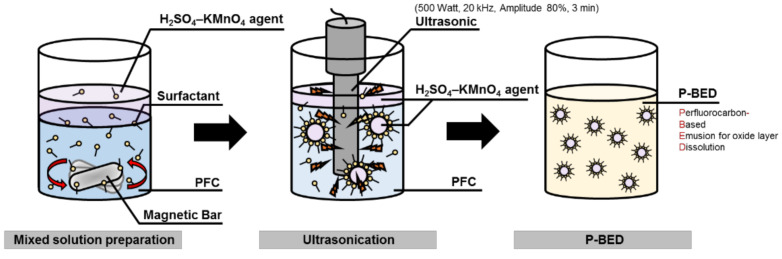
Preparation of P-BED.

The P-BED was prepared through the process, as represented in Figure 12. Firstly, PFC–surfactant–H_2_SO_4_ and KMnO_4_ agent mixed solutions were prepared. The solution was stirred at 500 rpm for 5 min using a magnetic stirrer (HSD 150-03P, MTOPS, Yangju-si, Gyeonggi-do, Korea,). After stirring, the ultrasonication process was applied to the mixed solution using ultrasonic (VC-505 Ultrasonic Processor, SONICS, Newtown, CT, USA). The decontamination agent in the PFC was dispersed homogeneously through this process. The application conditions of ultrasonic were 500 Watt of power, 20 kHz of frequency, and 80% of amplitude. The ultrasonication process was conducted for 3 min.

### 3.3. Dissolution Test

The dissolution performances of P-BED containing KMnO_4_ were evaluated using the simulated oxide layer of the SUS304 specimen. The specimen was prepared by a high-temperature oxidation process using a muffle furnace (MF-12, Hanyang Science Equipment Co., Nowon-gu, Seoul, Korea). The high-temperature oxidation process was conducted at 800 °C for 32 h. Before the oxidation process, the specimens were treated using 800 grit sizes of sandpaper. The simulated oxide layer mainly consisted of Cr_2_O_3_. The dissolution test was performed by immersing the simulated oxide layer specimen in the 50 mL of P-BED, while the P-BEDs were stirred at 250 rpm using a magnetic stirrer (MTOPS, HSD150-03P). The P-BEDs were stored in the bottle with the lid during the dissolution test. The temperature of P-BEDs was maintained at 45 °C. To compare the performance, the mass of the specimen was measured at 5, 10, 20, and 25 h, and the appearance change of the specimen was observed during the test. The weight loss was calculated using Equation (4).

Weight loss = (Initial specimen weight − specimen weight at time t) / (surface area of specimen), [g/m^2^] (4)

### 3.4. Analysis

The bonding characteristics of the P-BED were analyzed using FTIR (ABB, MB3000). The P-BED was filtered using a PTFE syringe filter (0.2 μm pore size, Whatman, Maidstone, Kent, UK) before the FTIR analysis. The morphology of the simulated oxide layer specimens before and after the high-temperature oxidation was observed using FE-SEM (Merlin Compact, ZEISS, Oberkochen, Land Baden-Württemberg, Germany). In addition, the composition of the simulated oxide layer specimens was analyzed by EDS (ZEISS, Merlin Compact, ZEISS, Oberkochen, Land Baden-Württemberg, Germany). During the dissolution, the weight change of the simulated oxide layer specimen was measured using the electronic micro-balance (XS204, Mettler Toledo, Columbus, OH, USA).

## 4. Conclusions

A number of metallic equipment installed in the nuclear facilities are contaminated with radionuclides contained in the oxide layer during the operation. The radionuclides have been generally removed by dissolving the oxide layer using the chemicals in an aqueous solution. It, however, generates a large amount of radioactive liquid waste as a secondary waste after dissolving the oxide layers and contaminants. Therefore, the removal method for dissolution of the oxide layer using reusable chemicals called P-BED is suggested in this study. P-BED consists of PFC, anionic surfactant, H_2_SO_4_, and an oxidant or the reducing agent. An advantage of P-BED is that the P-BED can be reused the PFC and surfactant after easily removing the used decontamination agent located at the top of the spent P-BED. This is because the emulsion medium is easily separated over time, and the used decontamination agent can be separated from the P-BED. They can be removed by scooping from the top of the solution because the density of the decontamination agent is lower than PFC. Therefore, the P-BED can be effectively used for decontamination of metal equipment, and it can contribute to reducing the amount of radioactive waste generation.

FTIR result of P-BED revealed that the homogenous dispersion of H_2_SO_4_ in the PFC was caused by the interaction between the carboxyl group of anionic surfactants, and the water including H_2_SO_4_. The results of the dissolution test showed that the simulated oxide layer specimen was satisfactorily removed by applying the P-BED method. During the dissolution of the oxide layer, the dispersion stability of the P-BED gradually decreased, and the H_2_SO_4_–KMnO_4_ was separated from the PFC. In addition, it was confirmed that the MnO_4_^−^ ions in the P-BED were consumed as participating in the dissolution reaction of Cr_2_O_3_. From the results, the behaviors of the P-BED during the dissolution of the oxide layer were illustrated. Based on the research result, it is possible to evaluate the oxide layer removal performance using the reused P-BED in the future.

## Figures and Tables

**Table 1:** EDS (at X1000) results of the specimen after a 25 h dissolution test under various concentrations of H_2_SO_4_ in P-BED.

Element	Atomic Percent (%)		
	0.37 M H_2_SO_4_ + 0.13 mM KMnO_4_	0.18 M H_2_SO_4_ + 0.13 mM KMnO_4_	0.00 M H_2_SO_4_ + 0.13 mM KMnO_4_
O	7.96	59.44	65.96
Cr	17.07	17.96	15.96
Fe	67.89	22.01	17.41
Ni	7.08	0.58	0.66
Total	100.00	100.00	100.00

**Table 2:** EDS (at X1000) results of the specimen after a 25 h dissolution test under various concentrations of KMnO_4_ in P-BED.

Element	Atomic Percent (%)		
	0.37 M H_2_SO_4_ + 0.00 mM KMnO_4_	0.37 M H_2_SO_4_ + 0.06 mM KMnO_4_	0.37 M H_2_SO_4_ + 0.13 mM KMnO_4_
O	44.17	32.97	7.96
Cr	16.23	9.80	17.07
Fe	37.98	54.09	67.89
Ni	1.61	3.15	7.08
Total	100.00	100.00	100.00

## Data Availability

Not applicable.
